# Molecular Design Principles for Photoactive Transition Metal Complexes: A Guide for “Photo-Motivated” Chemists

**DOI:** 10.1021/jacs.5c02096

**Published:** 2025-03-27

**Authors:** Giacomo Morselli, Christian Reber, Oliver S. Wenger

**Affiliations:** † Department of Chemistry, University of Basel, St. Johanns-Ring 19, 4056 Basel, Switzerland; ‡ Département de chimie, Université de Montréal, Montréal QC H3C 3J7, Canada

## Abstract

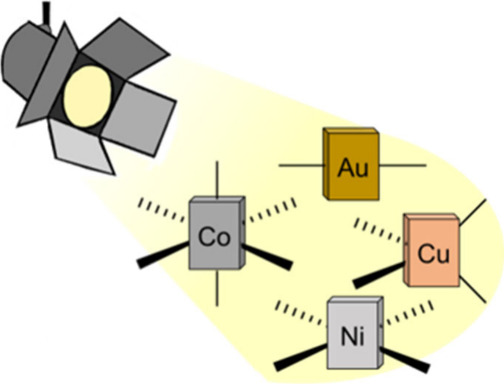

Luminescence and photochemistry involve electronically excited states that are inherently unstable and therefore spontaneously decay to electronic ground states, in most cases by nonradiative energy release that generates heat. This energy dissipation can occur on a time scale of 100 fs (∼10^–13^ s) and usually needs to be slowed down to at least the nanosecond (∼10^–9^ s) time scale for luminescence and intermolecular photochemistry to occur. This is a challenging task with many different factors to consider. An alternative emerging strategy is to target dissociative excited states that lead to metal–ligand bond homolysis on the subnanosecond time scale to access synthetically useful radicals. Based on a thorough review at the most recent advances in the field, this article aims to provide a concise guide to obtaining luminescent and photochemically useful coordination compounds with d-block elements. We hope to encourage “photo-motivated” chemists who have been reluctant to apply their synthetic and other knowledge to photophysics and photochemistry, and we intend to stimulate new approaches to the synthetic control of excited state behavior.

## Introduction

Chemists who are not involved in photophysics and photochemistry often are surprised that seemingly the same compounds are used over and over again for luminescence and photochemical applications. Conversely, in photophysics and photochemistry, we are envious of the much larger chemical space available to chemists interested in electronic ground state properties and thermally induced chemical reactivity. This contrast is based on a simple kinetic challenge: the energy temporarily stored in an electronically excited state is very quickly converted into molecular vibrations and heat if no measures are taken to prevent this. Here we provide an overview of these measures for coordination complexes of metals from the d-block of the periodic table and thus establish guidelines for obtaining luminescent and photochemically useful d-metal complexes. Luminescent complexes are used in a variety of applications, ranging from sensing and imaging to photonics and electroluminescent devices,^1−4^ while other applications involving the solar energy conversion and photocatalysis benefit from the enhanced redox properties of the excited states compared to the ground state.^5^ We will find that the set of factors controlling the decay of electronically excited states leads to certain privileged families of compounds, in which luminescence and useful photochemistry are more readily achievable than in others. However, we will also find that there are important exceptions and that with sufficient creativity and motivation, many important fundamental discoveries can still be made in the field of photoactive transition metal complexes.

## The Kinetic Challenge in Photophysics and Photochemistry

**Figure 1:**
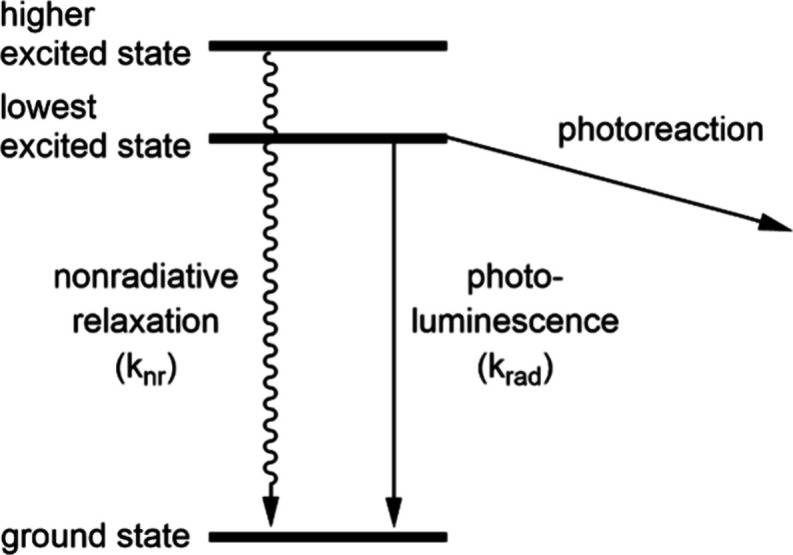
Energy-level diagram illustrating nonradiative relaxation, luminescence and photochemical reactivity from an electronically excited state.

Electronically excited states can generally decay via two competing channels (Figure 1): nonradiative relaxation (conversion of the excitation energy into molecular vibrations and heat) and radiative relaxation (luminescence). Both processes are characterized by first-order decay kinetics and rate constants (k_nr_, k_rad_) in units of s^–1^, and the natural lifetime (τ_0_) of the excited state is the reciprocal of the sum of these rate constants (τ_0_ = 1/(k_nr_ + k_rad_)). As a rule of thumb, an electronically excited state should have a natural lifetime τ_0_ of at least 1 ns to become luminescent and be used in intermolecular photochemistry.

As far as luminescence is concerned, the validity of this τ_0_ = 1 ns guideline is based in the fact that k_rad_ typically reaches maximum values close to 10^9^ s^–1^ for allowed electronic transitions. If k_nr_ can be limited to values below 10^8^ s^–1^ at the same time, very high luminescence quantum yields (ϕ) can be obtained, because ϕ = k_rad_/(k_nr_ + k_rad_).^6^

As far as intermolecular photochemistry is concerned, diffusion can define the limit for the required excited-state lifetime τ_0_. At room temperature, diffusion in many solvents proceeds with rate constants around (k_dif_) 10^9^ M^–1^ s^–1^, and assuming that a substrate is dissolved at a typical concentration (c_sub_) of 100 mM, the pseudo first order rate constant for a photochemical reaction with this substrate is k_dif_ × c_sub_ = 10^8^ s^–1^ (Figure 1). With a lifetime τ of 1 ns in the presence of the substrate, this means that the maximally achievable quantum yield ϕ_R_ for photochemical reaction with this substrate is ϕ_R_ = k_dif_ × c_sub_ × τ = 0.1, which implies that 1 in 10 excitations triggers a photoreaction. The first elementary step in a total substrate-to-product conversion triggered by light is usually followed by a series of light-independent steps with limited quantum yields; it is therefore helpful if ϕ_R_ is at least 0.1.^7^

The main finding so far is that lifetimes τ_0_ of at least 1 ns are desirable for both luminescence and intermolecular photochemistry. Longer lifetimes may be better to increase ϕ and ϕ_R_, but with limitations, as longer lifetimes also increase the likelihood of photodegradation.^8,9^ The main challenge in finding compounds with potential applications in photophysics and photochemistry is therefore to control k_rad_ and k_nr_. The remainder of this Perspective will largely focus on the different strategies for achieving this. For more fundamental aspects of photophysics and photochemistry, the reader is referred to previously published reviews and tutorials.^6,7^

## Overview of the Factors Controlling the Electronic Structure and Excited-State Decay

**Figure 2:**
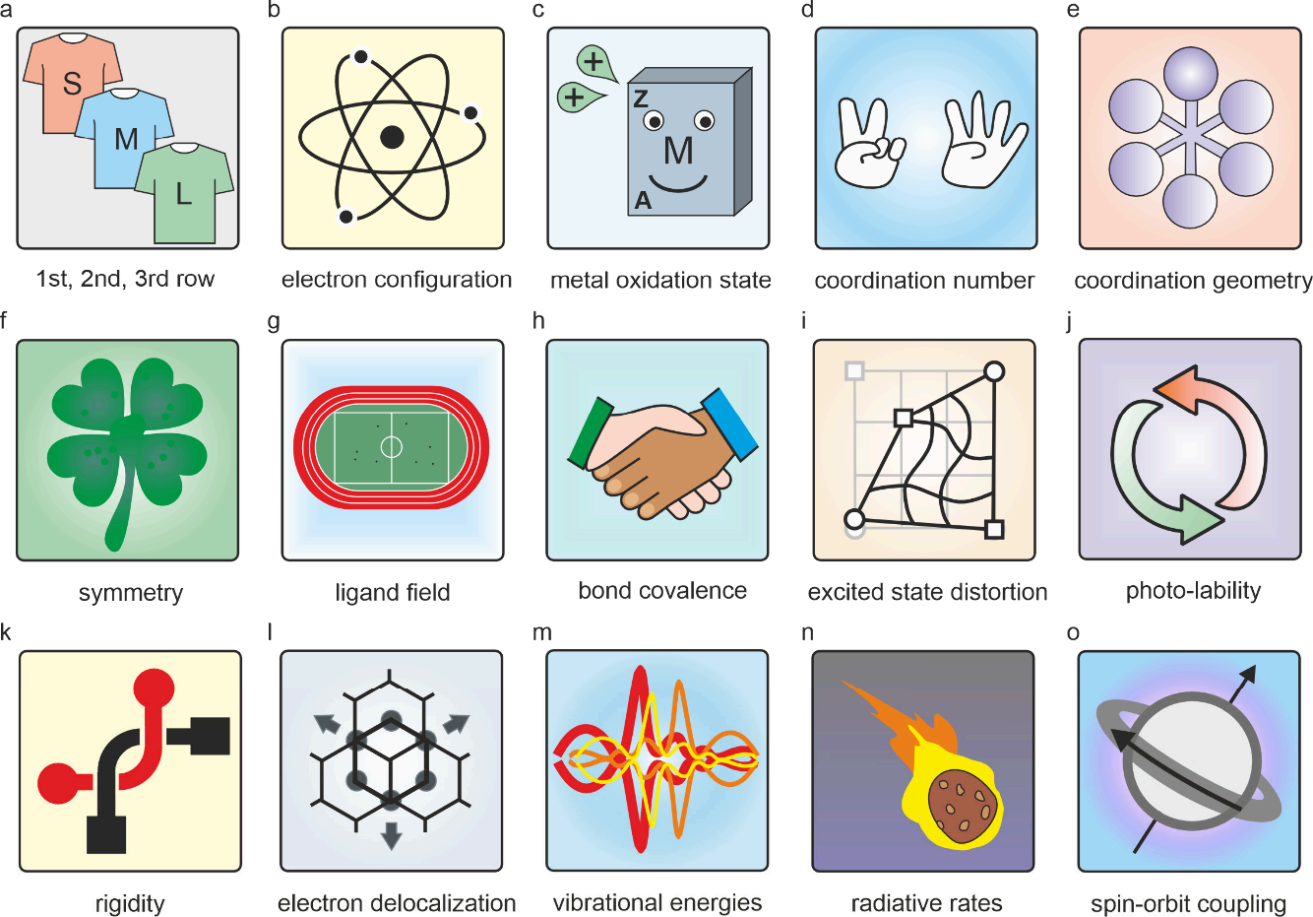
Factors controlling the molecular and electronic structures of photoactive metal complexes.

In this section, we first take a brief look at all relevant molecular design factors and go through Figure 2 square by square before discussing the individual factors separately in the following sections. Our search for photoactive transition metal complexes begins with the periodic table of elements and the choice of a metal from either the first, second or third transition series (Figure 2a). The “size” of the d-metal, more specifically how far the relevant 3d, 4d or 5d orbitals extend toward the coordinated ligands, is of paramount importance.^10^ This determines the strength of the metal–ligand interactions, which strongly influence the electronic structure of the complex. Equally important is the d-electron count, which determines the valence electron configuration of the metal (Figure 2b). For each d^n^ configuration, ligand field theory predicts a certain sequence of (metal-centered) electronically excited states, which makes it possible to rationally target various types of excited states. Third, the metal oxidation state (Figure 2c) is key, because it has a large influence on the redox potential of the metal and the tendency to form excited states with charge-transfer character. For a given d^n^ configuration, the metal oxidation state furthermore affects the d-orbital expansion. The coordination number (Figure 2d) and the geometry (Figure 2e) determine the symmetry (Figure 2f) of the metal complex, the combination of which influences the strength of the metal–ligand interactions, the energetic sequence of the excited states and the radiative (k_rad_) and nonradiative relaxation rates (k_nr_) of the excited states. The ligand field (Figure 2g) together with the metal–ligand bond covalence (Figure 2h), which depend on the “size” of the metal, its oxidation state, coordination number and coordination geometry, largely determine the energetic sequence and the difference between individual electronic states.

The redistribution of electron density in the excited state alters bond strengths and elongations between the metal and ligand, potentially causing significant distortion in the coordination environment while maintaining the overall geometry (e.g., octahedral, tetrahedral). Such excited-state distortions (Figure 2i) have a very strong influence on k_nr_ and on the ligand lability (Figure 2j) of the complex. The rigidity of the coordination sphere (Figure 2k) and the delocalization of excited electron density (Figure 2l) can counteract the distortion of the complex upon excitation. Molecular vibrations (Figure 2m) occur owing to the distortions of excited states, and their amplitudes and vibrational energies influence k_nr_. The spin–orbit coupling (Figure 2o) determines the exact angular momentum of the photoactive excited state(s) based on their spin multiplicity and orbital contributions and thus has a direct effect on k_rad_; in photochemical contexts it determines the type of reactivity to be expected. The radiative rates (Figure 2n) are adjustable by controlling the character of the luminescence transition, not only whether it is a spin-allowed transition or not, but also whether other selection rules are satisfied.

In the following individual sections, selected examples are used to show how the various design factors in Figure 2 can affect the photophysical and photochemical properties of metal complexes.

## Metal Atom “Size”

**Figure 3:**
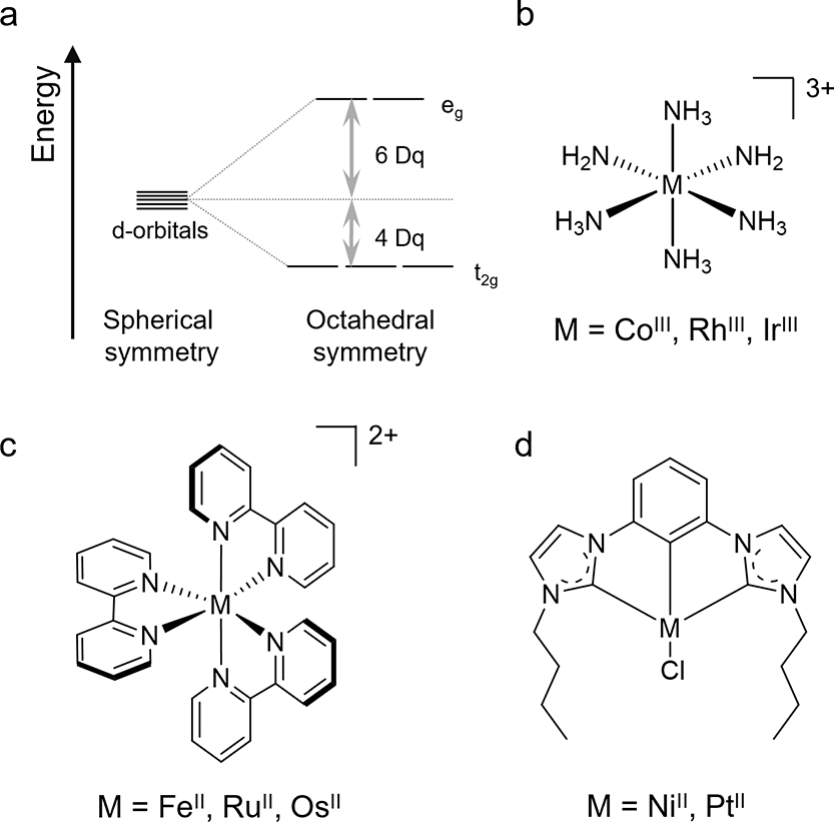
(a) Ligand-field splitting of d orbitals in octahedral symmetry.^11^ (b) Hexaammine complexes of group 9 trications. (c) Tris(2,2′-bipyridine) complexes of group 8 dications. (d) Square-planar complexes of group 10 dications: [Ni(dbyp)Cl] and [Pt(dbyp)Cl] (dpyb = 1,3-di(pyridyl)benzene).^13,14^

The d-orbitals usually contribute heavily to at least either the highest occupied molecular orbital (HOMO) or the lowest unoccupied molecular orbital (LUMO), and consequently the d-orbitals strongly influence the photophysical and photochemical behavior. The energies of the d-orbitals strongly depend on the interaction of the metal with its coordinated ligands. This interaction increases as one moves from the first to the second and third row of the transition series, because the radial distribution probability function of the d-electrons exhibits maxima further and further away from the metal nucleus when going from 3d to 4d and 5d orbitals.^10^ The homologous series of group 9 metal cations, including Co^III^, Rh^III^, and Ir^III^ in 6-fold ammonia ligand environments (Figure 3b), illustrates this effect.^11^ In such an octahedral coordination, the five d-orbitals split into a triply degenerate set of π- or essentially nonbonding t_2g_ orbitals and a doubly degenerate set of σ-antibonding e_g_ orbitals (Figure 3a).^11^ The ligand field parameter (10 Dq), which captures the energy difference between t_2g_ and e_g_ orbitals, increases by about 50% from [Co(NH_3_)_6_]^3+^ to [Rh(NH_3_)_6_]^3+^ and by a further 20% to [Ir(NH_3_)_6_]^3+^ (Table 1).^11^ The metal-nitrogen bond lengths in these complexes increase from 1.96 Å for Co^III^ to 2.07 Å for Rh^III^ and 2.09 Å for Ir^III^.^12^ The key point is that first-row transition metal complexes generally experience substantially weaker ligand fields than second- or third-row transition metal complexes.^10^

**Table 1:** Photophysical Parameters in d^6^ and d^8^ Metal Complexes from the 1st, 2nd, and 3rd Row of the Transition Series in Solution at Room Temperature

	10 Dq/cm^–1^		τ/ns	ϕ	λ_em_/nm		τ/ns	ϕ	λ_em_/nm
[Co(NH_3_)_6_]^3+^	23,300^a^	[Fe(bpy)_3_]^2+^	5 × 10^–5^	0	N/A	[Ni(dpyb)Cl]^d^	9 × 10^–4^	0	N/A
[Rh(NH_3_)_6_]^3+^	34,400^a^	[Ru(bpy)_3_]^2+^	855^b^	0.095^b^	621				
[Ir(NH_3_)_6_]^3+^	41,200^a^	[Os(bpy)_3_]^2+^	60^c^	0.005^c^	746	[Pt(dpyb)Cl]^d^	7200	0.6	491

a From ref (11).

b In deaerated CH_3_CN.^15,16^

c In deoxygenated CH_3_CN.^17^

d In CH_2_Cl_2_.^13,14^ τ is the lifetime of the lowest MLCT excited state.

Cations of group 8 metals, including Fe^II^, Ru^II^, and Os^II^ in 2,2′-bipyridine (bpy) ligand environments (Figure 3c) illustrate the positive effect of stronger ligand fields in second- and third-row transition metal complexes. [Ru(bpy)_3_]^2+^ and [Os(bpy)_3_]^2+^ have largely metal-centered HOMOs and ligand-based LUMOs, leading to lowest excited states with strong metal-to-ligand charge transfer (MLCT) character, which are luminescent and redox-active (Table 1).^18^ On the contrary, in [Fe(bpy)_3_]^2+^ the ligand field is much weaker and two metal-centered (MC) excited states involving t_2g_ – e_g_ electron promotions are below the MLCT state,^19^ shortening the MLCT lifetime to 50 fs and leading to the loss of the photoluminescence, a property that MC states often do not have (Table 1).^20,21^ The situation is similar for metals with other electron configurations and different coordination numbers and geometries, for example in square-planar d^8^ complexes (Figure 3d): [Pt(dbyp)Cl] luminesces with a quantum yield of 0.6 from a mixed ligand-centered (LC, i.e. a transition between orbitals mainly localized on the ligands) and MLCT excited state with a lifetime of 7.2 μs,^13^ while in [Ni(dbyp)Cl] the lowest MLCT state decays to an MC excited state within 0.9 ps (Table 1).^14^ More comprehensive comparisons of the photophysics of Ni^II^, Pd^II^ and Pt^II^ complexes were made in the solid state.^22^

## Electron Configuration

**Figure 4:**
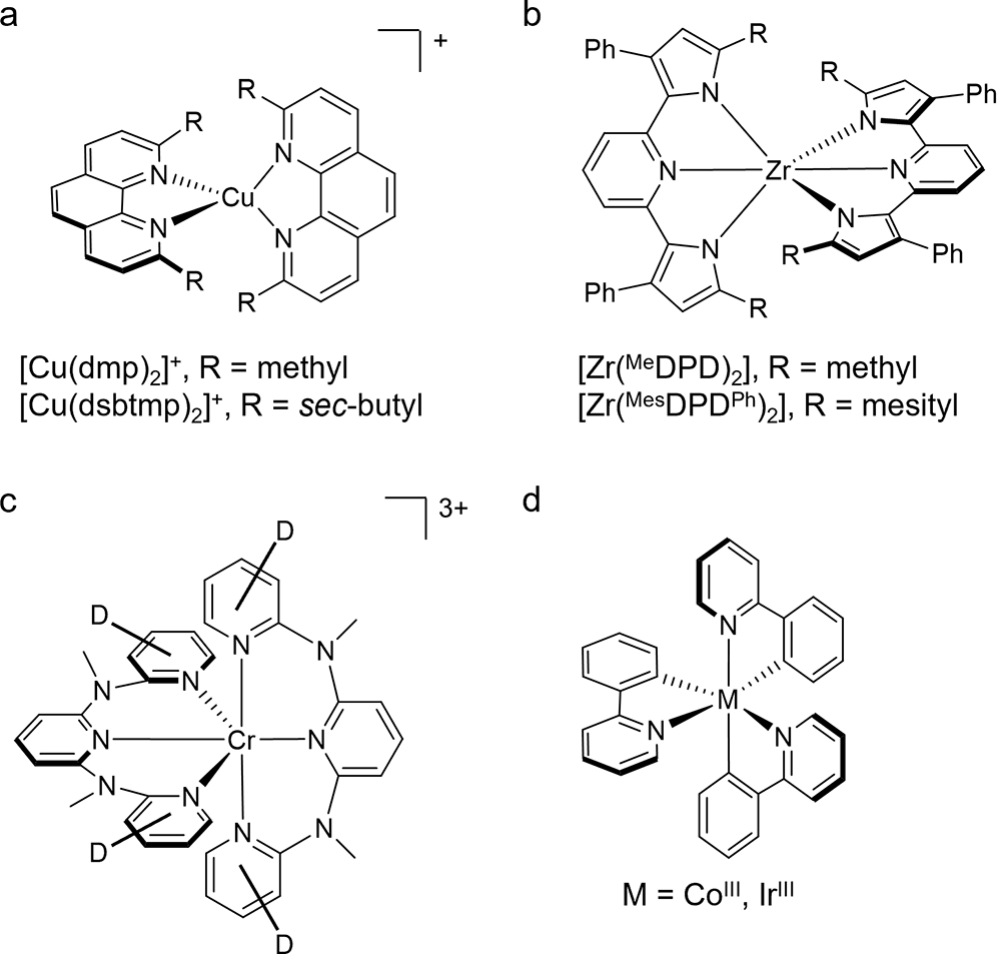
(a) [Cu(dmp)_2_]^+^ (dmp = 2,9-dimethyl-1,10-phenanthroline)^24,25^ and [Cu(dsbtmp)_2_]^+^ (dsbtmp = 2,9-di(*sec*-butyl)-3,4,7,8-tetramethyl-1,10-phenanthroline).^24^ (b) [Zr(^Me^PDP)_2_]^27^ and [Zr(^Mes^PDP^Ph^)_2_].^28^ (c) [Cr([D_9_]-ddpd)_2_]^3+^ (ddpd = N,N′-dimethyl-N,N’-dipyridine-2-yl-pyridine-2,6-diamine).^32^ (d) *fac*-[Ir(ppy)_3_] and *fac*-[Co(ppy)_3_] (ppyH = 2-(phenyl)pyridine).^33,34^

Some d-electron configurations are clearly favored over others when aiming at long-lived excited states. The d^0^ and d^10^ configurations are especially easy to work with, because there are no low-energy MC states, as the d-orbitals are either empty of fully occupied. The deactivation of charge-transfer (CT) excited states by MC states described in the previous section (Figure 3) thus no longer arises. This is a key reason for the existence of a very large number of Cu^I^ complexes with MLCT and related CT excited states, which exhibit good photoluminescence and photocatalytic properties.^23−25^ Tetrahedral Cu^I^ complexes (Figure 4a) face other challenges leading to unwanted nonradiative deactivation, in particular molecular distortions toward square-planar geometry in the MLCT excited state, which limits their photoluminescence quantum yields and lifetimes. These distortions can be limited (see below) so that MLCT lifetimes in the microsecond range and ϕ values in the order of 0.05 are achieved in solution at room temperature (Table 2).

**Table 2:** Photophysical Parameters of Selected Metal Complexes with Privileged d-Electron Configurations in Solution at Room Temperature

Configuration	Complex	τ/μs	ϕ	λ_em_/nm
d^10^	[Cu(dmp)_2_]^+^^a^	0.09	2.1 × 10^–4^	670
d^10^	[Cu(dsbtmp)_2_]^+^^a^	2.8	0.063	631
d^8^	[Pt(dbyp)Cl]^a^	7.2	0.60	491
d^0^	[Zr(^Me^PDP)_2_]^b^	325	0.08	594
d^0^	[Zr(^Mes^PDP^Ph^)_2_]^b^	350	0.45	581
d^3^	[Cr([D_9_]-ddpd)_2_]^3+^^c^	2300	0.30	778
d^6^ low-spin	*fac*-[Ir(ppy)_3_]^d^	4.0	0.97	508

a In CH_2_Cl_2_.^13,24,25^

b In THF.^27,28^

c In CD_3_CN.^32^

d In 2-methyl-THF.^33^ λ_em_ denotes the emission band maximum.

In d^0^ complexes, metals in high oxidation states are typically combined with electron-rich ligands, so that ligand-to-metal charge transfer (LMCT, i.e. an electronic transition between an orbital mainly localized on the ligand to an orbital mainly localized on the metal) excited states are most important.^26^ The [Zr(^Me^PDP)_2_] and [Zr(^Mes^PDP^Ph^)_2_] complexes (Figure 4b) illustrate this design principle and photophysical behavior (Table 2),^27,28^ as do, for example, recently reported Ti^IV^, Re^II^ and W^VI^ complexes.^29−31^

In octahedral coordination environments, the d^3^ valence electron configuration is also one of the most privileged, since the lowest excited state in this situation can be a so-called “spin-flip” with very low molecular distortions, almost like f-f excitations in lanthanides.^35^ Achieving high photoluminescence quantum yields and long excited-state lifetimes is therefore inherently easier than in other cases where the d-subshell is only partially filled. The recently developed “molecular ruby” family of compounds (Figure 4c) featuring ϕ values up to 0.30 for a deuterated complex and a 2.3 ms lifetime illustrates these favorable circumstances (Table 2).^32^

The low-spin d^6^ configuration that is typically achieved in octahedral complexes with strong ligand fields is perhaps the most frequently targeted scenario, but this configuration is only privileged for the second and the third row of the transition series typically comprising precious metals (see above).^18,36^ One of the best performers is *fac*-[Ir(ppy)_3_] (Figure 4d, Table 2).^33^ Square-planar d^8^ complexes are somewhat similar to the low-spin d^6^ scenario because electron-rich metals provide access to photoactive MLCT states and because this behavior is largely restricted to second- and third-row transition metals such as Pt^II^ and Au^III^ (Figure 3d, Table 1).^37^

All other d-electron configurations are not preferred for photoluminescence and photocatalysis (Table 3). This includes the case of low-spin d^5^ in octahedral coordination environments, for which Fe^III^ complexes were recently discovered by chance.^38,39^ There could of course be other, as yet undiscovered, cases in which a particular configuration and coordination environment is considered poorly suited, but favorable molecular design results in low-lying MC states being less detrimental than anticipated.^40^

**Table 3:** Preferred and Non-preferred Valence Electron Configurations of Molecular d-Metal Complexes for Luminescence and Photocatalytic Applications in Solution at Room Temperature

Ground state configuration	Coordination geometry	Classification	Typical photoactive excited states
d^0^, d^10^	octahedral, tetrahedral	privileged	CT (LMCT for d^0^, MLCT for d^10^) and LC
d^6^ (low-spin)	octahedral	privileged for 4d and 5d metals	MLCT
d^8^ (low-spin)	square planar	privileged for 4d and 5d metals	MLCT, LC
d^3^	octahedral	privileged for strong-field ligands	MC (spin-flip)
d^2^, d^4^, d^8^	octahedral	somewhat privileged for strong-field ligands	MC (spin-flip)
d^5^ (low-spin)	octahedral	somewhat privileged for Fe^III^ carbenes	LMCT
d^1^, d^7^, d^9^	octahedral	not privileged	

To conclude this section, we note that the type of electronic excitation made (CT, LC or MC) influences the penetration depth of the excitation light and the local density of molecules in the excited state, which can be important for photocatalysis.^41,42^ The molar absorption coefficient (ε) of (spin-allowed) CT and LC transitions are high (often > 10^4^ M^–1^ cm^–1^), whereas MC transitions usually display substantially lower ε values (10–10^3^ M^–1^ cm^–1^).

## Metal Oxidation State

**Figure 5:**
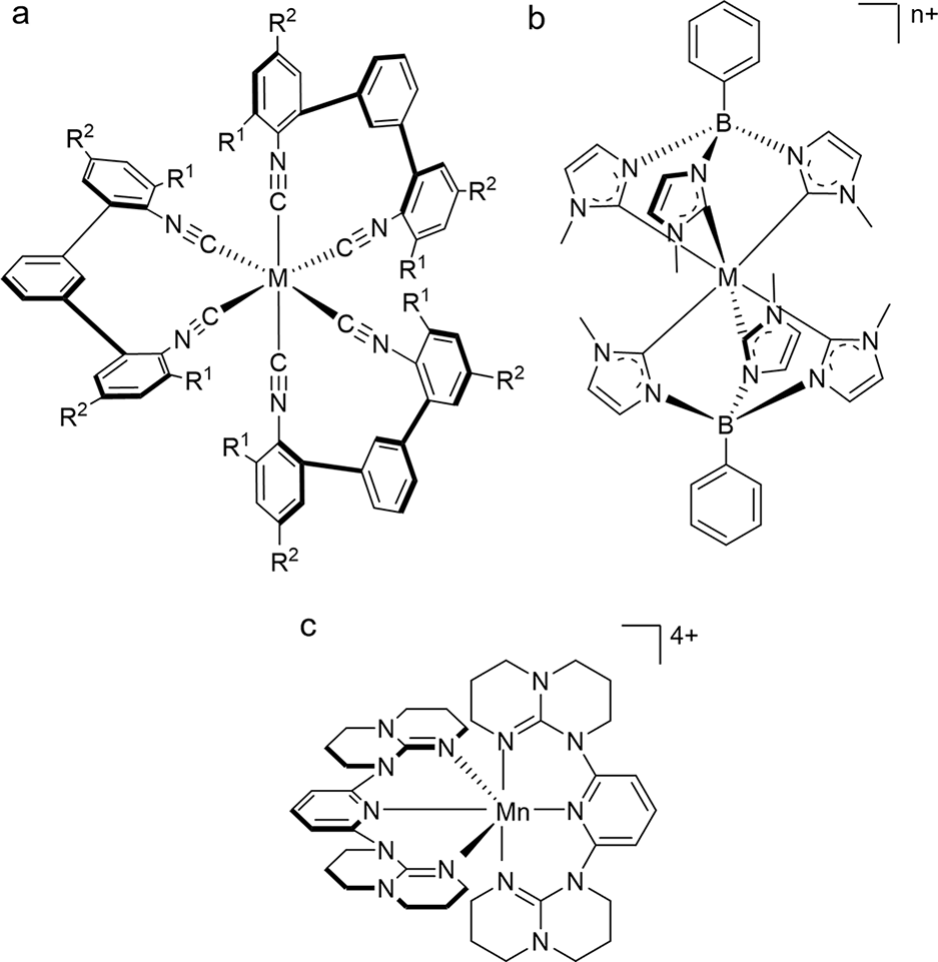
(a) Tris(diisocyanide) complexes of M = Cr^0^, Mn^I^ and Fe^II^ (R^1^ = *tert*-butyl, R^2^ = pyrenyl).^44^ (b) Fe^III^ (*n* = 1) and Mn^IV^ complexes (*n* = 2) with identical LMCT energies.^39,48^ (c) [Mn(dgpy)_2_]^4+^ (dgpy = 2,6-diguanidylpyridine).^49^

Both the metal–ligand bonding and the redox properties of a coordination complex are strongly influenced by the oxidation state of the metal. Consequently, within a given d-electron configuration, the energies of MC, MLCT and LMCT excited states are all significantly affected by the oxidation state of the metal. Isocyanide coordination environments illustrate this particularly well, as they can stabilize a wider range of oxidation states than other ligands.^43^ In three isostructural and isoelectronic isocyanide complexes (Figure 5a), the energy of the lowest MLCT excited state increases by about 1.0 eV between Cr^0^ and Mn^I^ and by at least 0.5 eV between Mn^I^ and Fe^II^ (Table 4),^44^ reflecting the stabilization of the metal-based HOMO with increasing nuclear charge. Similarly pronounced shifts in MLCT energies are observed between isostructural and isoelectronic Fe^II^ and Co^III^ polypyridines.^45^ The more difficult the metal is to oxidize, the higher the MLCT energy.

**Table 4:** Influence of Metal Oxidation State (Within a Given d Electron Configuration) on the Energy of Some of the Key Excited States

Configuration	Metal	Complex	Type of the excited state	Energy of the excited state/eV	10 Dq/cm^–1^
3d^6^ low-spin	Cr^0^	[Cr(^bi^CNR^Pyr^)_3_]^a^	MLCT	∼1.6	
3d^6^ low-spin	Mn^I^	[Mn(^bi^CNR^Pyr^)_3_]^+^^a^	MLCT	∼2.6	
3d^6^ low-spin	Fe^II^	[Fe(^bi^CNR^Pyr^)_3_]^2+^^a^	MLCT	≥3.1	
3d^6^ low-spin	Fe^II^	[Fe(bpy)_3_]^2+^	MC		21,000^b^
3d^6^ low-spin	Co^III^	[Co(bpy)_3_]^3+^	MC		25,900^b^
d^3^	Cr^III^	[Cr(CN)_6_]^3-^	LMCT	>4.0^c^	
d^3^	Mn^IV^	[Mn(CN)_6_]^2-^	LMCT	3.2^c^	

a In THF.^44^

b Ligand-field splitting parameter 10 Dq indicated here instead of the energy of the lowest MC excited-state; the lowest excited state is different for Fe^II^ and Co^III^ in these cases.^45,51^

c In CH_3_CN.^46,47^ The ligand-field splitting parameters are determined at the MC absorption band maxima and therefore refer to the complexes that are still in their ground state geometry, while the other excited state energies given here reflect those of the relaxed excited state geometries.

Conversely, the energies of the LMCT excited states decrease with increasing metal oxidation state, as shown, for example, in the comparison of [Cr(CN)_6_]^3-^ and [Mn(CN)_6_]^2-^ (Table 4), where the first LMCT transition occurs at an energy more than 1.0 eV lower for Mn^IV^ compared to the isoelectronic Cr^III^.^46,47^ Such comparisons are only valid for metals with the same electron configuration, as shown by Fe^III^ and Mn^IV^ complexes with identical hexa(carbene) coordination environments, where the energy of the lowest LMCT excited state is essentially identical (Figure 5b).^39,48^ In this case, there is not only a change in metal oxidation state but also a change from the d^5^ low-spin to the d^3^ electron configuration, so that this comparison is not entirely valid. Similarly, the direct comparison of the same complex with its metal in different oxidation states, as is possible for [Mn(dgpy)_2_]^4+^ (Figure 5c), for example, changes many factors at once.^49^

The energies of MC excited states are very sensitive to the oxidation state of the metal. In most, but not all cases, MC state energies increase with increasing metal oxidation states, and the resulting changes in electronic structure are best captured by the ligand field parameter (10 Dq).^50^ This parameter is a measure of the ligand field strength and increases by more than 15% when going from Fe^II^ to Co^III^ in the same polypyridine coordination environment (Table 4).^45,51^ As the oxidation state of the metal increases, the covalent character of the metal–ligand bonds tends to increase, which also has an effect on the MC state energies (see below).^52^

Most photoactive complexes have metals in the +II or +III oxidation states, as this is usually a good compromise between stability and reactivity. Lower oxidation states can be very reducing in photocatalysis, but it can become difficult to close catalytic cycles as it becomes difficult to reduce the oxidized complexes back to their initial state.^53^ Higher oxidation states can lead to very strongly oxidizing photochemistry, which poses similar challenges and sometimes causing oxidation of solvents.^54^ MC and CT excited states can exhibit similar photoinduced electron transfer behavior,^55^ but there is evidence that (Dexter-type) energy transfer from shielded MC excited states can be slower than that of complexes with CT excited states. This feature has been elegantly exploited to obtain an oxygen-tolerant molecular ruby luminophore that achieves a remarkably long spin-flip MC excited state lifetime of 0.5 ms in aerated solution at room temperature.^56^

## Coordination Number, Geometry and Symmetry

**Figure 6:**
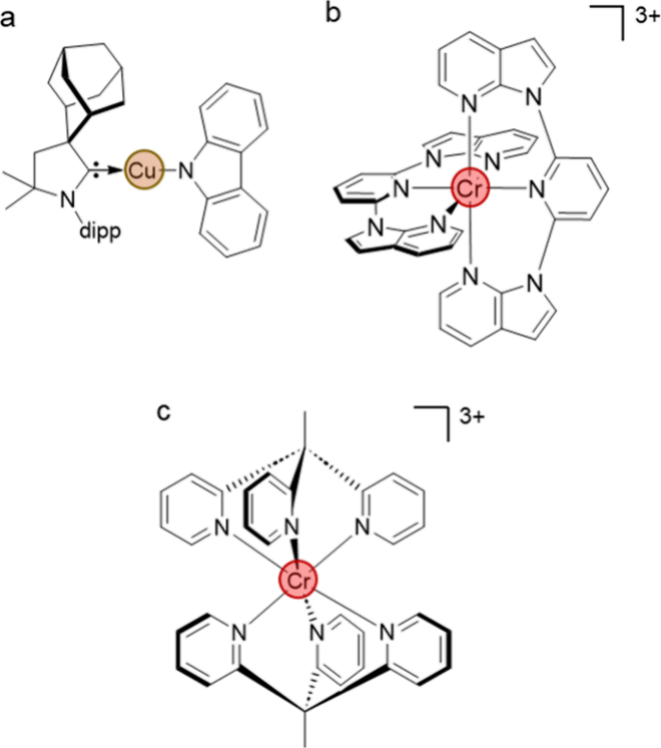
(a) Cu^I^ complex with a cyclic(alkyl)(amino)carbene (CAAC) ligand and an N-bound amide ligand; dipp = 2,6-diisopropylphenyl.^57^ (b) [Cr(dNinp)_2_]^3+^ (dNinp = 2,6-di(*N*-7-azaindol-1-yl)pyridine).^64^ (c) [Cr(tpe)_2_]^3+^ (tpe = 1,1,1-tris(pyrid-2-yl)ethane).^65^

The most important coordination numbers in photoactive d-metal complexes are 6, 4 and 2. Six-coordinate octahedral complexes with the electron configurations d^0^, d^3^, d^5^ low-spin or d^6^ low-spin are most frequently investigated. The ligand fields of octahedral complexes are more than twice as strong as those of tetrahedral complexes,^11^ which is advantageous for eliminating low-energy MC states that could cause efficient nonradiative deactivation of excited states. Such low-energy MC states cannot occur in d^0^ and d^10^ complexes, which is why most photoactive complexes with a tetrahedral coordination environment have these configurations.^23^ With 4d^8^ and 5d^8^ metals and also with 3d^8^ metals in strong ligand fields (e.g., with CO, CN^−^ or isocyanides), four-coordinate complexes are square-planar, since this geometry strongly stabilizes the d_z2_ orbital, while the d_x2-y2_ orbital can remain unoccupied.^11,37^ Linear two-coordinate complexes with photoactivity in solution are less common, but can be obtained with d^10^ metals.^57−59^ Complexes with high photoluminescence quantum yields (ϕ > 0.5) in solution have been reported mainly for octahedral 5d^6^ complexes containing Ir^III^ (Figure 4d, ϕ = 0.97),^33,60,61^ square planar d^8^ complexes of Pt^II^ (Figure 3d, ϕ = 0.60)^13,62^ and recently also for two-coordinate d^10^ complexes including Cu^I^ (Figure 6a, ϕ = 1.0).^57^ Four-coordinate tetrahedral complexes, especially with Cu^I^, perform less well in this respect, as considerable molecular distortions occur in their MLCT excited states (see below).^24^ Six-coordinated octahedral complexes play a dominant role in photocatalysis. Due to the ligand substitution inertness of octahedral d^6^ low-spin and d^3^ complexes, they therefore are well suited for outer-sphere electron transfer and for triplet sensitization.^5^ Conversely, the ligand lability of four-coordinated Cu^I^ compounds offers opportunities for substrate coordination in photocatalysis.^23,63^

The majority of photoactive metal complexes do not exhibit the idealized symmetries described above. Heteroleptic ligand coordination environments can allow independent tuning of HOMO and LUMO energies,^60,66^ and the combination of electron-rich with electron-poor ligands on the same metal can be useful to obtain photoactive CT excited states (Figure 6a).^57^ Symmetry lowering also affects the d-orbital energies and can cause orbitals that are degenerate in the O_h_, T_d_ or D_4h_ point groups to become energetically different.^67^ In the tris(2,2′-biypridine) complexes of Figure 3c, for which the actual point group is D_3_, this effect is small, but in other cases the resulting sequence of MC excited states with slightly different energies can become an effective nonradiative decay channel. In cases where symmetry lowering occurs because some metal–ligand bonds are substantially longer than others, nonradiative relaxation is also favored due to the readily accessible large excited state distortions. For example, in the [Cr(dNinp)_2_]^3+^ complex (Figure 6b), the Cr^III^–N distances to the central pyridine are ca. 3% longer than the Cr^III^–N distances to the azaindole units, and the luminescence lifetime of this compound is ca. 0.87 μs,^64^ 3 orders of magnitude shorter than for (nondeuterated) [Cr(ddpd)_2_]^3+^ (0.9 ms),^32^ where the longest and the shortest Cr^III^–N distances differ by only about 1% (Figure 4c). Similar observations were also made for Ru^II^ complexes.^68^ A set of intensely luminescent, six-coordinate Cr^III^ complexes has been nicknamed as molecular rubies,^35^ in analogy to the strongly emitting corundum derived mineral where a small percentage of Cr^3+^ substitutes Al^3+^. Emission is due to the lowest energy spin-flip transition in both types of materials. The family of molecular ruby compounds owes the long lifetime of its luminescent excited states in large part to its high symmetry. In [Cr(tpe)_2_]^3+^ (Figure 6c), where inversion symmetry is present, this leads to a particularly long lifetime of 4.5 ms for the photoactive excited state.^65^

## Ligand Field Strength

In complexes of metals with a partially filled d-subshell, the ligand field is important regardless of the nature of the photoactive excited state. The term “ligand field” is primarily associated with a certain symmetry, which defines the resulting electronic structure qualitatively in a generalizable fashion for all d electron configurations, the Tanabe-Sugano diagrams.^50^ In this section, we deal with more quantitative aspects by focusing on the strength of the ligand field in a particular symmetry.

The metal has the strongest influence on the strength of the ligand field, as discussed in the sections on metal atom size and oxidation state. When going from 3d to 4d and 5d metals, the energetic splitting between t_2g_ and e_g_ orbitals (Figure 3a), which is described by the ligand field parameter (10 Dq) in octahedral symmetry, increases, since d electrons with larger spatial extent interact more strongly with the ligands. Higher oxidation states also strengthen the ligand field due to reinforced interactions with the Lewis basic ligands.

**Figure 7:**
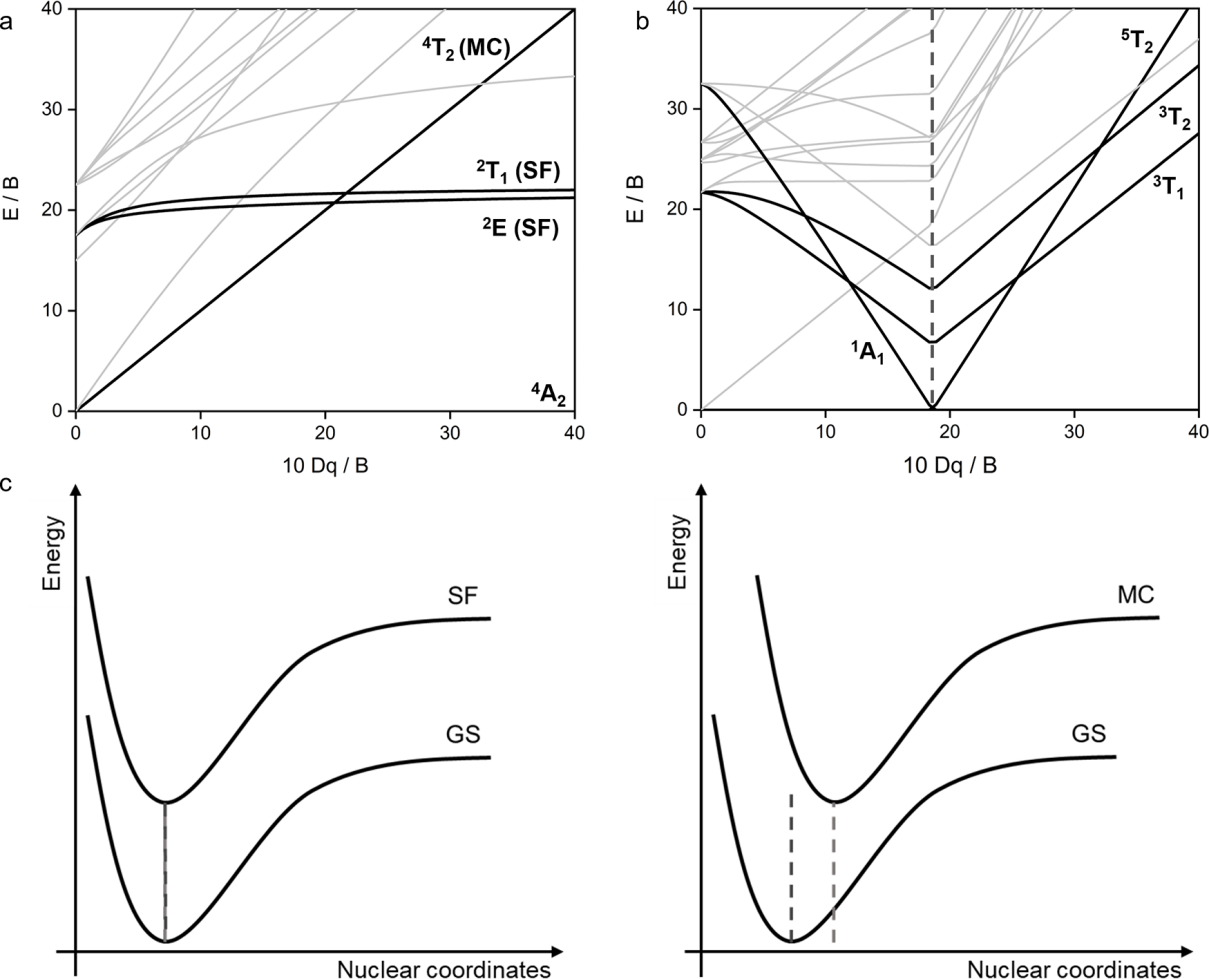
Tanabe–Sugano diagram for (a) octahedral d^3^ and (b) octahedral d^6^ complexes.^50^ (c) Single configuration coordination diagrams illustrating the different geometric distortions of spin-flip (SF) and ligand field-dependent metal centered (MC) excited states relative to the ground state. GS denotes the ground state.

The influence of the ligands is determined by their binding properties. Essentially all ligands are σ-donors, but some of them also have π-donor properties, for example halide anions. π-donor ligands destabilize the t_2g_ orbitals of the metal (Figure 3a) and therefore cause weaker ligand fields and lower 10 Dq values.^11,18^ In the octahedral [CrCl_6_]^3-^ coordination unit (embedded in a crystalline host lattice of Cs_2_NaYCl_6_), ^4^T_2_, in which the promotion of a t_2g_ electron to an e_g_ orbital has taken place, becomes the lowest excited state (Figure 7a).^69^ Conversely, π-acceptor ligands stabilize the t_2g_ orbitals and lead to stronger ligand fields and higher 10 Dq.^11,18^ Consequently, in [Cr(ddpd)_2_]^3+^ the lowest excited state is the ^2^E state,^32^ in which all three d electrons have remained in the t_2g_ orbitals, but one of the electron spins is flipped with respect to the ^4^A_2_ ground state. This change of the photoactive excited state has drastic effects on the photophysical and photochemical behavior (see next section) and therefore allows the properties to be tuned.

Similar changes in the lowest excited MC state are also possible in other d-electron configurations, e.g. in the low-spin d^6^ configuration, in which a change from a ^5^T_2_ to a ^3^T_1_ as the lowest excited state occurs between Fe^II^ and Co^III^ in polypyridine coordination (Figure 7b).^51^ This change fundamentally alters the decay behavior of the excited states and leads to a situation in Co^III^ similar to that of noble metals, where the nonradiative relaxation slows down with increasing energy of the excited state.^70^ This and the strongly oxidizing character of the Co^III 3^T_1_ excited state opens new possibilities for photocatalysis.^71^

Tuning the energy of excited MC states by changing the ligand field strength can also be very effective when the photoactive excited state has MLCT, LMCT or LC character. In Fe^II^ polypyridines, the ^5^T_2_ and ^3^T_1_ MC states cause rapid nonradiative deactivation of the ^3^MLCT excited state which luminesces in [Ru(bpy)_3_]^2+^.^21^ MC state tuning in Co^III^ complexes is currently receiving increasing attention, with the goal of achieving luminescence properties or further lifetime prolongation.^72,73^

Enhancement of the ligand field with N-heterocyclic and mesoionic carbene ligands, which have better σ-donor and π-acceptor properties,^74,75^ shifts these MC states to higher energies and lead to an increase in the lifetime of the excited ^3^MLCT state from 50 fs in [Fe(bpy)_3_]^2+^ (Figure 3c) to 0.5 ns the best case.^76^ The excited-state lifetime of ^3^MLCT is further extended in isoelectronic Mn^I^ and Cr^0^ complexes with chelating arylisocyanide coordination environments (Figure 5a), even leading to ^3^MLCT photoluminescence.^53,77^ The strong ligand fields of the isocyanide ligands are helpful in shifting MC states to higher energies, while the lower metal oxidation states stabilize the luminescent ^3^MLCT states.^78^

## Metal–Ligand Bond Covalence

**Figure 8:**
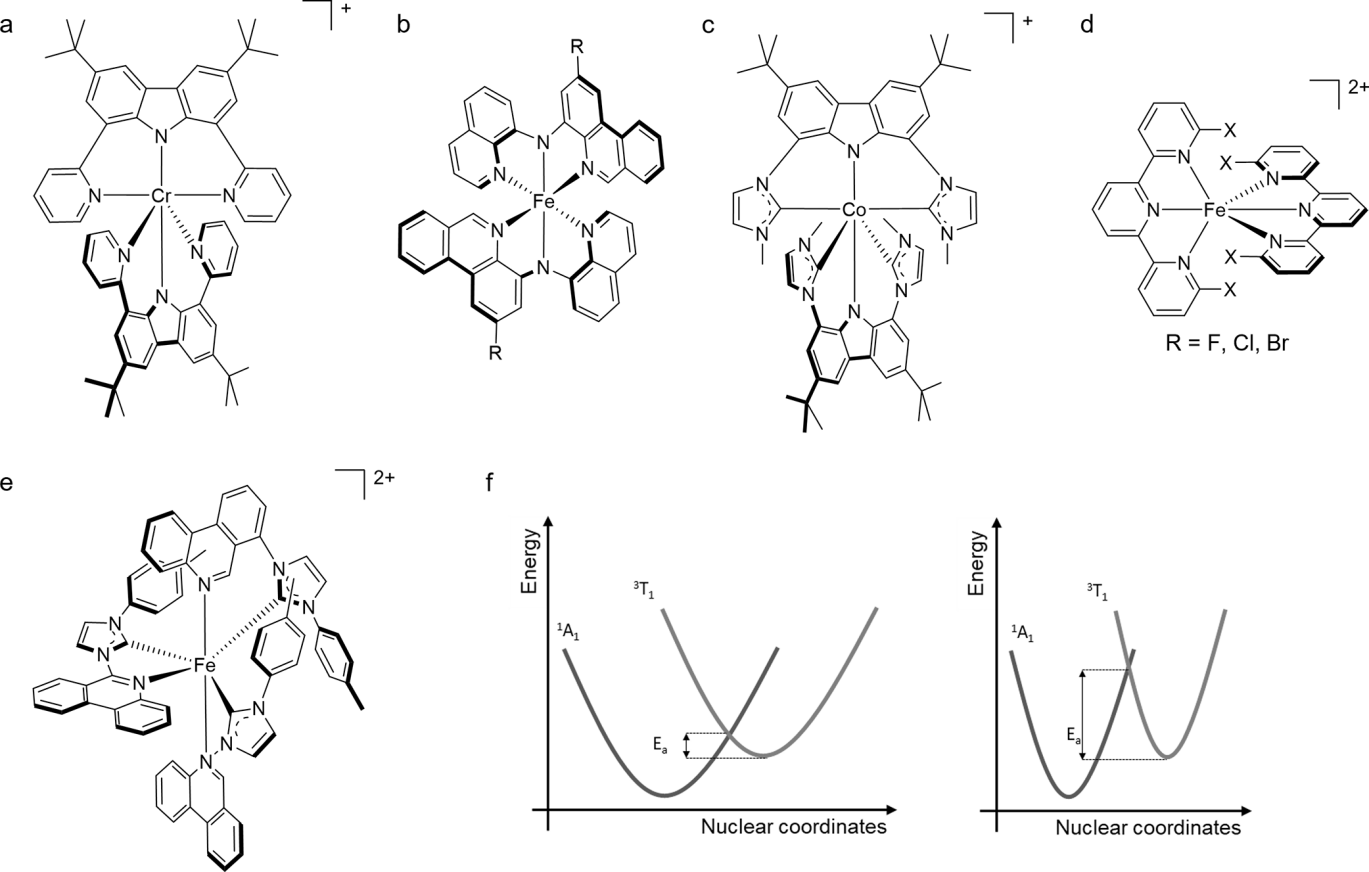
(a) [Cr(dpc)_2_]^+^ (dpc = 3,6-di-*tert*-butyl-1,8-di(pyridine-2-yl)-carbazolato).^79^ (b) [Fe(pqa^R^)_2_] (R = ^*t*^Bu, CF_3_, Cl; pqa = (phenanthridin-4-yl)(quinoline-8-yl)amido).^85−87^ (c) [Co(bimca)_2_]^+^ (bimca = 1,8-bis(imidazoline-2-yliden-1-yl)carbazolide).^88^ (d) High-spin [Fe(dftpy)_2_]^2+^ (dftpy = 6,6″-difluoro-2,2′:6′,2″-terpyridine), [Fe(dctpy)_2_]^2+^ (dctpy = 6,6″-dichloro-2,2′:6′,2″-terpyridine) and [Fe(dbtpy)_2_]^2+^ (dbtpy = 6,6″-dibromo-2,2′:6′,2″-terpyridine) complexes with ^5/7^MLCT excited states.^89,90^ (e) [Fe(L)_3_]^2+^ (L = 1-(5λ^4^-phenanthridin-5-yl)-3-(p-tolyl)-1*H*-imidazol-3-ium-2-ide).^91^ (f) Potential energy surfaces of the ground ^1^A_1_ and excited ^3^T_1_ states for an octahedral low-spin d^6^ complex in the absence (left) and in the presence (right) of the rigidifying effect caused by intramolecular π-stacking.

Cr^III^ polypyridine complexes luminesce from a ^2^E state (see above), which is independent of the ligand field strength (Figure 7a), since it is a spin-flip excited state in which no electron promotion takes place between different orbitals.^35^ As a result, Cr^III^ polypyridines glow in the low-energy red or near-infrared spectral region with comparatively small variations in the luminescence band maximum.^79^ Changing the ligand field strength is ineffective for tuning the luminescence wavelength, instead the covalency of the metal–ligand bond becomes an important controlling factor, even if it is not yet quantitatively understood.^52,80^ The amido ligand of [Cr(dpc)_2_]^+^ is a strong π-donor (Figure 8a), leading to significantly more covalent Cr^III^-N bonds than in the case of pyridine units.^79^ This covalency is quantified by the Racah B parameter, which measures the repulsion between d electrons on the metal,^11^ and this B value decreases from about 700 cm^–1^ in Cr^III^ polypyridines to about 500 cm^–1^ in [Cr(dpc)_2_]^+^.^79^ Since the energy of the ^2^E spin-flip state is a direct function of B (Figure 7a),^81^ this leads to a drastic decrease of the ^2^E_g_ energy in [Cr(dpc)_2_]^+^ and luminescence at 1067 nm.^79^ Several subsequent studies followed this design principle and then achieved luminescence at room temperature beyond 800 nm in molecular Cr^III^ complexes.^52,82,83^

More covalent metal–ligand bonds can also generally be obtained by increasing the metal oxidation state.^52^ For example, in a Mn^IV^ complex with carbene ligands (Figure 5b), the ^2^E luminescence is substantially red-shifted compared to isoelectronic Cr^III^ complexes due to the increasing covalency.^84^

Covalency is an underestimated shaping factor of photoactive metal complexes compared to the strength of the ligand field, although it is clear that the MC state energies are determined by the ratio of 10 Dq and B (Figure 7a/b).^50^ This simple quantitative relationship was exploited in Fe^II^ complexes with amido π-donor ligands (Figure 8b) to achieve higher 10 Dq/B ratios than in comparable Fe^II^ polypyridines by allowing an overall favorable tradeoff between decreasing ligand field strength (lower 10 Dq) and increasing covalency (lower B). As a result, the lowest MC excited state was shifted to significantly higher energies compared to the charge-transfer (CT) excited states,^85^ but unfortunately not as much as originally expected,^86^ leading to a reassignment of the lowest excited state in this class of compounds to an MC instead of a CT state.^87^

Cyclometalated complexes such as *fac*-[Ir(ppy)_3_] are another important class of compounds, where covalency can lead to complications in determining the character of the photoactive excited state. The M-C bonds tend to be more covalent than the M-N bonds, and it can become difficult to distinguish between LC and MLCT excited states.^92^ Covalency can be used to tune the CT state energies,^93^ as shown by the example of the compound [Co(bimca)_2_]^+^ (Figure 8c),^88^ where the photoactive excited state has considerable MLCT character due to the much higher electron density of the formal Co^III^ cation compared to Co^III^ polypyridines.^45^

At present, the dependence of the Racah B parameter on molecular structure is less quantitatively understood than the relationship between molecular structure and the ligand field parameter 10 Dq.^52^ One reason for this may be that it is often less straightforward to obtain reliable information about the B parameter than about 10 Dq.

## Excited-State Distortion, Photolability, Rigidity, Electron Delocalization

The redistribution of electron density associated with electronic excitation inevitably changes the bonding situation in a metal complex. Depending on the metal and its electron configuration, the coordination geometry, the ligand field and the exact nature of the electronic transition, this can lead to a more or less strong distortion of the molecular complex in the excited state.

Cu^I^ diimine complexes (Figure 4a) are preferably tetrahedral in their electronic ground states.^24^ Their lowest excited state is often of the MLCT type, with the metal oxidized to Cu^II^, which prefers five- or six-coordinate environments. Consequently, Cu^I^ diimine complexes have a strong tendency to coplanarize their chelating ligands and bind additional ligands (for example solvent molecules) in the MLCT state.^94^ To limit these distortions and improve the MLCT luminescence lifetime and quantum yield, the concept of cooperative rigidity was introduced, which essentially consists of preventing the coplanarization of the chelating ligands by sterically demanding substituents (Figure 4b).^95^

**Figure 9:**
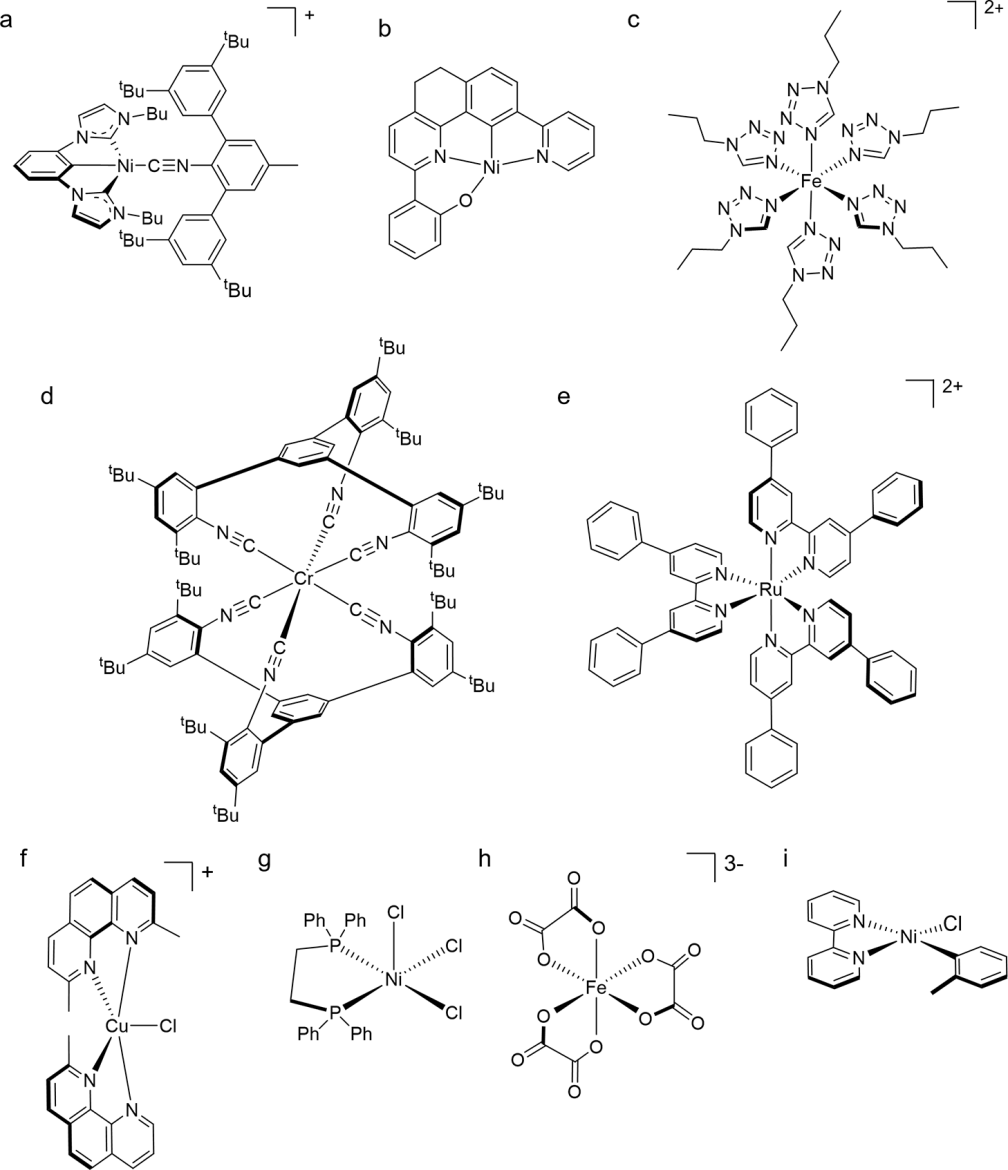
(a) Ni^II^ complex with a sterically demanding isocyanide ligand to limit the distortion from square-planar to tetrahedral geometry.^97^ (b) Ni^II^ complex with a rigid tetradentate ligand.^99^ (c) The [Fe(ptz)_3_]^2+^ spin-crossover compound (ptz = 1-propyltetrazole).^102,103^ (d) Cr^0^ complex undergoing reversible photoinduced dissociation of an arylisocyanide coordination unit.^9^ (e) [Ru(dpb)_3_]^2+^ (dpb = 4,4′-diphenyl-2,2′-bipyridine).^104^ (f) [Cu(dmp)_2_Cl]^+^ as a precursor to the [Cu(dmp)_2_]^+^ complex.^105^ (g) [NiCl_3_(dppe)] (dppe = bis(diphenylphosphino)ethane).^106^ (h) The ferrioxalate complex. (i) [Ni(dtbpy)(*o*-tolyl)Cl] (dtbpy = 4,4′-di-*tert*-butyl-2,2′-bipyridine) as an exemplary Ni^II^ complex undergoing VLIH.^107^

Ni^II^ forms square-planar complexes in strong ligand fields (Figure 3d), which tend to relax within picoseconds into MC states with tetrahedral geometry upon MLCT excitation.^96^ Cooperative rigidity can be exploited to prevent this distortion and achieve ^3^MLCT lifetimes up to about 50 ps (Figure 9a).^97,98^ An alternative approach is to use tetradentate ligands with increased rigidity (Figure 9b).^99^ Other current Ni^II^ work focuses on obtaining fluorescence from covalently attached organic moieties, but this is a very different approach from trying to control the inherent photophysical properties of the metal complex.^100,101^

Although these Cu^I^ and Ni^II^ complexes do not necessarily undergo bond breaking reactions, they nevertheless represent extreme cases of molecular distortions in electronically excited states. At the other extreme are octahedral Cr^III^ complexes with photoactive spin-flip (^2^E) excited states (Figure 7a), in which the bonding situation remains largely unchanged because only an electron spin is changed. This forms the basis for the very slow nonradiative relaxation in the class of molecular ruby compounds (Figure 4c/6c) and represents an inherent advantage over other classes of photoactive complexes. However, the reverse intersystem crossing to the energetically higher ^4^T_2_ state (Figure 7a) must be prevented because the ^4^T_2_ state is highly distorted, which can lead to photoinduced ligand dissociation.^108^

The equivalents of the ^4^T_2_ state in the d^3^ configuration are the ^5^T_2_ and ^3^T_1_ excited states in the low-spin d^6^ configuration (Figure 7b), as these states all involve the promotion of electrons from d orbitals, which are to a first approximation nonbonding (t_2g_ in Figure 3a), to antibonding d orbitals (e_g_ in Figure 3a).^18^ The ^5^T_2_ state in particular is strongly distorted, with the Fe-N distances in Fe^II^ complexes such as [Fe(ptz)_3_]^2+^ (Figure 9c) lengthening by up to 0.2 Å.^102,103^

The ^3^T_1_ state relevant in isoelectronic Co^III^ complexes is less distorted,^51^ but photoinduced (partial) ligand dissociation can nonetheless occur even in chelate complexes such as *fac*-[Co(ppy)_3_] (Figure 4d).^34^ The same MC-excited states are responsible for the photoinduced release of carbon monoxide from isoelectronic Mn^I^ complexes with the [Mn(CO)_3_] motif,^109^ many of which are of interest for the light-triggered and targeted release of CO for therapeutic purposes.^108^ Mn^I^ complexes with monodentate isocyanide ligands (isolobal to CO) undergo the same type of ligand photodissociation,^110^ and chelating aryl isocyanide ligands have been developed to prevent this (Figure 5a).^77^ In a Cr^0^ isocyanide complex with two facially coordinating tridentate ligands, photoinduced reversible dissociation of an arylisocyanide coordination unit was recently observed (Figure 9d).^9^ In contrast, Mo^0^ complexes are very robust in similar coordination environments and provide record photoluminescence quantum yields.^111^ This illustrates the (photo)substitution-inertness of second-row transition metal complexes in comparison to first-row transition metal complexes.^112^

In Fe^II^ bis(tpy) complexes (tpy = 2,2′:6′:2″-terpyridine), halogen substituents in α-position to the coordinating N-atoms of the peripheral pyridines (Figure 8d) restrict the conformational degrees of freedom.^89,90^ The MLCT lifetime increases from 14.0 to 16.0 and 17.4 ps as one moves from F to Cl to Br, consistent with the view that greater steric hindrance leads to less nonradiative excited state relaxation. However, in this series of compounds, the repulsive interactions between the halogen atoms of one tpy ligand and the other coordinated tpy are strong enough to weaken the ligand field to the extent that a high spin d^6^ valence electron configuration results. This is in contrast to the vast majority of photoactive d^6^ metal complexes, which have a low-spin configuration.

Intramolecular π-stacking between individual ligands can also be exploited to enhance rigidity, as demonstrated recently in an Fe^II^-NHC complex with phenanthridine ligands (Figure 8e). The resulting narrower potential energy curves in both the ^1^A_1_ ground and ^3^T_1_ excited states (Figure 8f), here again in the low-spin d^6^ configuration, lead to a higher activation barrier for nonradiative relaxation of the excited state to the ground state.^91^ Similar findings were reported earlier for thiocyanate and selenocyanate complexes of Pd^II^ and Pt^II^ upon applied pressure.^113^

The MLCT-excited states of octahedral low-spin d^6^ metal complexes are less distorted than these ligand-field-dependent MC states, but they are more distorted than the prominent spin-flip (ligand-field-independent) excited states of Cr^III^ complexes (^2^E in Figure 7a/c).^104^ Even small distortions are sufficient to promote the nonradiative relaxation of excited states.^114^ To counteract this, delocalization of the MLCT-excited electron over as much of the ligand periphery as possible in Ru^II^ polypyridine complexes bearing additional phenyl groups (Figure 9e) has been shown to be useful,^104,115,116^ and later this concept was extended to the luminescent MLCT-excited state of Cr^0^ complexes by the addition of pyrene groups (Figure 5a).^44,53^

The luminescent LMCT excited states of d^0^ complexes (Figure 4b) can be similarly weakly distorted as the MLCT excited states of low-spin d^6^ complexes,^28^ but for Fe^III^ (d^5^), Ni^III/II^ (d^7^, d^8^), Cu^II^ (d^9^), and several other types of complexes, the LMCT excited states can lead to photolability that triggers synthetically useful reactions.^117,118^ During LMCT excitation of penta-coordinated Cu^II^-chlorido complexes (Figure 9f), electron transfer from the chloride ligand to Cu^II^ takes place, leading to the release of a chlorine radical and tetrahedral Cu^I^ complexes.^105^ This visible light induced homolysis (VLIH) thus becomes a suitable method to form photoactive Cu^I^ complexes (Figure 4a) from more stable Cu^II^ precursors, and to release halogen atoms.^119^ Similar photoreactivity was directly observed in a Ni^III^ complex (Figure 9g).^106^ The dissociative character of the excited LMCT state is the key property that enables “LMCT catalysis”, in which reactive radicals are released and then undergo further chemical reactions in the ground state.^117^ Homolysis of metal–ligand bonds can occur on an ultrafast time scale, so that in these particular cases nanosecond excited state lifetimes are not required.^26^ For this reason, many Fe^III^ compounds, including simple salts such as Fe(NO_3_)_3_ or Fe_2_(SO_4_)_3_, are well suited for triggering LMCT catalysis,^120,121^ especially with carboxylate ligands, which upon LMCT excitation transform into unstable carboxyl radicals that decompose to CO_2_ and synthetically utilizable organic radicals.^122^ Conceptually, the first elementary steps after photoexcitation are similar to those of the well-known ferrioxalate complex (Figure 9h).^26^

In Ni^II^ complexes (Figure 9i), similar VLIH reactions occur and are relevant for nickel-based cross-coupling catalysis.^107,123^ The corresponding Ni–C homolysis reactions occur even from higher electronic excited states and thus do not obey Kasha’s rule,^107^ according to which only the lowest excited state (of a given spin multiplicity) is photoreactive.^124^

## Vibrational Energies and Radiative Rates

Even the most rigid molecule has vibrations that can dissipate electronic excitation energy. The higher the energy of a molecular vibration, the more effective it is in deactivating excited states.^17^ Because of the high vibrational frequencies of the C-H stretching modes (about 3000 cm^–1^), the ubiquitous C–H groups play a key role. In the [Cr(bpy)_3_]^3+^ complex, the ^2^E luminescence band overlaps with the fourth overtone of the aromatic C–H vibrations, whereas in [Cr(ddpd)_2_]^3+^ (Figure 4c) there is a spectral overlap mismatch between the luminescence and the C–H overtone absorption.^32^ These circumstances contribute to the much higher luminescence quantum yield and ^2^E lifetime of [Cr(ddpd)_2_]^3+^ compared to [Cr(bpy)_3_]^3+^.^32^ In most other transition metal complexes, the luminescent excited states are considerably more distorted than the spin-flip excited states of Cr^III^ emitters, and therefore such spectral overlap mismatches between vibrational overtones are less likely to be achievable. A more general strategy is to use deuteration,^125^ which can be very effective because the C-D stretching frequency is only about 2400 cm^–1^. The lower frequency resulting from the increased effective mass can reduce k_nr_. In [Cr(ddpd)_2_]^3+^, deuteration increases the luminescence quantum yield from 0.11 to 0.30 and the ^2^E lifetime from 0.9 to 1.3 ms, with partial deuteration being sufficient to achieve a large effect and the C-H/D bonds closest to the metal being most important.^32^ In [Ru(bpy)_3_]^2+^, complete deuteration results in only a 10% increase the ^3^MLCT lifetime,^126^ illustrating that deuteration does not generally lead to a drastic improvement.

Slowing down nonradiative relaxation events helps to extend excited state lifetimes, but luminescence quantum yields can be further optimized by accelerating radiative relaxation. For lighting applications, rapid depopulation of the excited state is desirable to minimize photodegradation, and photocatalysis can also benefit from lifetimes that are long enough to allow diffusion-controlled reactions with substrates, but short enough to minimize unwanted side reactions.^127^ Therefore, it may be desirable to increase the probability of radiative transitions by molecular design.

Large gaps between the lowest excited state and the electronic ground state are advantageous for luminescence, whereas small energy gaps favor nonradiative deactivation according to the energy gap law.^17^ This simplistic law describes the relationship between the nonradiative rate constant *k*_*nr*_ and the energy gap *ΔE* between the levels involved as an exponential function. Based on the energy gap law, it is easy to see why red or near-infrared emitting compounds usually have lower photoluminescence quantum yields.

**Figure 10:**
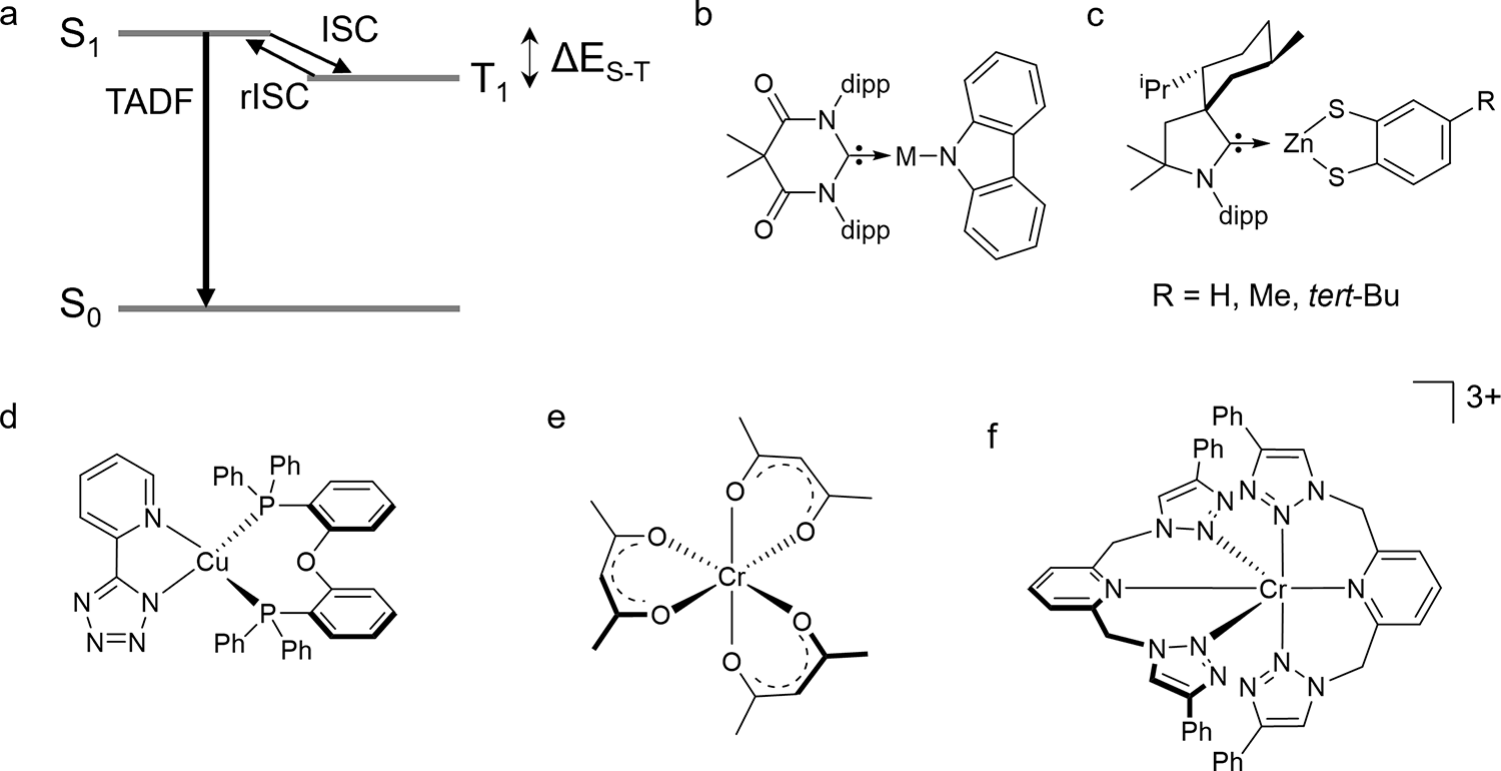
(a) Energy-level scheme illustrating TADF (rISC = reverse intersystem crossing). (b) Cu^I^, Ag^I^ and Au^I^ MAC complexes with small S_1_ – T_1_ energy gaps (MAC = 1,3-bis(2,6-diisopropylphenyl)-5,5-dimethyl-4-keto-tetrahydropyridylidene; dipp = 2,6-diisopropylphenyl).^128^ (c) Zn^II^ dithiolate complex showing TADF.^129^ (d) Cu^I^ complex undergoing ISC with a time constant of 27 ps.^130^ (e) [Cr(acac)_3_] (acac = acetylacetonate).^131^ (f) Cr^III^ triazole complex.^132^

Small energy gaps between the lowest singlet (S_1_) and triplet (T_1_) excited states can be helpful because they can lead to thermal repopulation of the fluorescent S_1_ state from the slower emitting T_1_ state (Figure 10a) in a phenomenon called thermally activated delayed fluorescence (TADF).^133^ The extended lifetime of the photoactive S_1_ state benefits photocatalysis by facilitating diffusion-controlled reactions,^134^ and TADF finds applications in photonic devices such as light-emitting electrochemical cells and organic light-emitting diodes.^135,136^ Small singlet–triplet energy gaps (ΔE_ST_) on the order of 0.3 eV or less are achievable for charge-transfer excited states in which the donor and acceptor units are well separated spatially so that the spin–spin interactions between the unpaired electrons of the S_1_ and T_1_ states are relatively similar.^137^ This can be achieved, for example, in many recently explored d^10^ complexes containing Cu^I^, Ag^I^, Au^I^ and Zn^II^ (Figure 10b/c).^57,128,129,138,139^

An alternative to the thermal repopulation of fast-emitting singlet excited states from long-lived triplets is to rely entirely on spin-allowed fluorescence-type transitions in metal complexes. The recently reported Fe^III^ scorpionate complex family (Figure 5b) owes its comparatively high photoluminescence quantum yields (0.017–0.019) in large part to the spin-allowed character of its LMCT emission,^39,140^ in contrast to the classical spin-forbidden MLCT luminescence of d^6^ and d^8^ metal complexes (Figure 3c/d) and also to the spin-forbidden LMCT luminescence of Zr^IV^ compounds (Figure 4b). The low radiative rate constants for the ^2^E → ^4^A_2_ spin-flip transition (Figure 7a/c) in Cr^III^ polypyridines limit the luminescence quantum yields of molecular rubies.^81^

## Spin–Orbit Coupling

Spin–orbit coupling is the interaction between an electron’s spin and its orbital angular momentum. Its strength increases approximately with the fourth power of the atomic number (Z^4^), making it small in light atoms but significant in heavy elements. In systems with weak spin–orbit coupling, spin and orbital contributions remain largely separable, allowing the definition of pure spin states (e.g., singlets and triplets). However, in heavy-atom-containing molecules, strong spin–orbit coupling leads to mixing of spin states, making a strict spin multiplicity distinction less meaningful. This mixing increases the efficiency of otherwise spin-forbidden processes, such as intersystem crossing and phosphorescence.

Light absorption is most effective for transitions leading to excited states with the same spin multiplicity as the ground state, i.e., singlet-to-singlet transitions for ground state species without unpaired electrons. Hund’s rule then predicts an energetically more stable triplet excited state (T_1_), which can be populated by intersystem crossing (ISC) from the initially excited singlet state (S_1_). The reactivity of S_1_ and T_1_ can be fundamentally different, and while S_1_ stores more energy for a short time, T_1_ stores less energy for a usually much longer time. The efficiency of ISC is strongly related to the spin–orbit coupling, and the spin–orbit coupling constants of individual chemical elements depend on the fourth power of the atomic number.^11^ Consequently, the spin–orbit coupling constants of first-row transition metals are considerably smaller than those of second- and third-row metals. Nevertheless, intersystem crossing often remains very fast in first-row transition metal complexes, but with some notable differences.

In *fac*-[Ir(ppy)_3_] (Figure 4d), ISC occurs within less than 100 fs and in [Fe(bpy)_3_]^2+^ (Figure 3c) similar sub-100 fs kinetics have been determined,^21^ whereas in a tetrahedral Cu^I^ complex (Figure 10d), ISC is significantly slower with a time constant of 27 ps.^130^ Apparently, there are large differences in ISC rates among first-row transition complexes that do not simply correlate with the magnitude of the spin–orbit coupling constant of a given metal element. Work on [Cr(acac)_3_] (Figure 10e)^131^ and a Cr^III^ triazolyl complex (Figure 10f)^132^ provides a more nuanced picture, in which the rate of intersystem crossing depends strongly on the types of electronic excited states involved (CT versus MC) and how strongly the respective electronic states are coupled.^131^ This coupling depends on the distortion of the relevant states relative to each other and their energy difference, with large distortions and small energy differences accelerating ISC.

The rate of ISC also appears to be particularly high when the spin change is coupled to an orbital change, for example between an excited ^1^LC state and a ^3^CT state, as in two-coordinate Cu^I^ complexes (Figure 6a).^57,128^ This behavior is similar to that described by the El-Sayed rules for organic carbonyl compounds.^6,141^ Clearly, the control of ISC rates and efficiencies depends on a complex interplay of different molecular design factors.

## Some Simple Recipes

Based on the considerations in the previous sections, we arrive at the following set of simple guidelines for the design of photoactive d-metal complexes.

- Aim for one of the electron configurations in Table 3, depending on the type of photoactive excited state you wish to achieve. Note that there may be important exceptions to these general guidelines, such as the recently discovered luminescent Fe^III^ complexes with excited state lifetimes ranging from 100 ps to 100 ns.^38,39,142^
- Create large energy gaps between the lowest excited state and the electronic ground state to counteract nonradiative relaxation. In situations where this is not possible, focus on an excited state characterized by a low geometric distortion, such as charge-transfer or spin-flip metal-centered excited states.
- In complexes with partially filled d-subshells, strong ligand fields combined with high metal–ligand bond covalence are helpful. Six-coordinate octahedral complexes are better for obtaining strong ligand fields than four-coordinate tetrahedral complexes. Use second or third row transition metals instead of first row transition metals to maximize ligand field strength. Use high metal oxidation states for strong ligand fields and high metal–ligand bonding covalence. In ligand design, find the right balance between obtaining strong ligand fields (e.g., with π-acceptor properties) and strong metal–ligand binding covalence (e.g., with π-donors, which tend to induce weak ligand fields). Note that the exact metal/ligand combination is important, as polypyridines are π-acceptors to second and third row transition metals, but can become π-donors to first-row transition metals.^45^
- Minimize molecular distortions in the photoactive excited state relative to the ground state by using stiff chelate ligands, rigidifying the coordination sphere around the metal, and ensuring delocalization of the excited electron density. Complexes with high symmetry are usually a better starting point for achieving this than complexes with large distortions already in the electronic ground state.
- Bi- and tridentate chelating ligands are helpful in mitigating the effects of photolability and limiting unwanted photodegradation.
- Avoid C–H groups and other high energy oscillators in close proximity to the metal. Consider deuteration or introduction of other heavier atoms to lower vibrational energies.
- To enhance luminescence, accelerate radiative excited state relaxation by making the electronic transition between the lowest excited state and the ground state more allowed, e.g., by targeting spin-allowed transitions or by exploiting the concept of thermally activated delayed fluorescence.

## Conclusions and Outlook

The rational design of d-metal coordination compounds with luminescent and photochemically useful excited states requires the consideration of a complex interplay of design factors, but the available chemical space is becoming increasingly large. Excited state lifetimes and luminescence quantum yields can be increased by tailoring coordination environments, opening up possibilities beyond the traditionally explored platinum group metal compounds.^143^ The limits of what is possible through synthetic control of excited state properties do not appear to have been reached yet, and an even greater diversity of photoactive d-metal complexes than has been established so far is realistically achievable. Exploring these limits through combined (synthetic) innovations in coordination and organometallic chemistry, photophysics, and photochemistry seems important from a fundamental research perspective. While simple coordination spheres and readily available ligands remain attractive for many applications, innovations beyond the traditional ligand classes and metal species have led and are likely to continue to lead to groundbreaking new photophysical and photochemical insights.

Conventional photoredox reactions often rely on a collisional encounter between the excited metal complex and a reaction partner in an outer-sphere electron transfer. Alternatively, coordinated ligands may transfer electrons to the metal in an inner-sphere electron transfer. Under such conditions, the kinetic limitations outlined in the introductory part of this paper no longer apply. Inner-sphere electron transfer reactions involving substrates coordinated as ligands to metals have opened up opportunities for synthetic organic chemistry, often involving metal complexes that would *a priori* be considered unsuitable for photochemistry due to apparently too short excited state lifetimes.^117,144^ The dissociative nature of these excited states allows reactivity on subnanosecond timescales, sometimes from higher excited states.^145^ How far such fast photochemistry can be extended beyond metal–ligand bond homolysis reactions and inner-sphere electron transfer is an important open question.^146^

Academic research is becoming increasingly application-oriented, and finding the right balance between curiosity-driven research and delivering a marketable product is becoming more and more challenging. Research on first-row photoactive transition metal complexes illustrates this dilemma to some extent. On the one hand, it is argued that first-row transition metals are much more abundant and cheaper than platinum group metals,^147,148^ but many of the ligands needed to obtain luminescent and photoredox-active first-row transition metal complexes require considerably more synthetic work (and therefore are more expensive) than the long-known ligands used for photoactive noble metal complexes. However, the knowledge gained from the development of conceptually new ligands and photoactive complexes could be very valuable for advancing the fields of photophysics and photochemistry as a whole. With this in mind, we hope that our article can encourage chemists from different specializations and backgrounds to contribute their synthetic and other knowledge to the development of fundamentally new types of photoactive d-metal complexes.
